# Effect of Rumen-Protected Lysine Supplementation on Growth Performance, Blood Metabolites, Rumen Fermentation and Bacterial Community on Feedlot Yaks Offered Corn-Based Diets

**DOI:** 10.3390/ani15192901

**Published:** 2025-10-04

**Authors:** Yan Li, Yuzhong Chen, Peng Wu, Abraham Allan Degen, Kelei He, Qianyun Zhang, Xinsheng Zhao, Wanyu Li, Aiwen Zhang, Jianwei Zhou

**Affiliations:** 1 State Key Laboratory of Herbage Improvement and Grassland Agro-Ecosystems, College of Pastoral Agriculture Science and Technology, Lanzhou University, Lanzhou 730020, China; liyan20251115@163.com (Y.L.); zhaoxinsheng1997@126.com (X.Z.); liwy2024@lzu.edu.cn (W.L.); 2 Gansu Extension Station of Animal Husbandry Technology, Lanzhou 730030, China; 3 Hezuo Workstation of Animal Husbandry, Hezuo 747000, China; m13884062072@163.com (Y.C.); m15209406942@163.com (K.H.); 4 Hangzhou King Techina Feed Company, Ltd., Hangzhou 311107, China; wupeng_kdq@126.com (P.W.); zhangqianyun_kdq@126.com (Q.Z.); 5 Desert Animal Adaptations and Husbandry, Wyler Department of Dryland Agriculture, Blaustein Institutes for Desert Research, Ben-Gurion University of the Negev, Beer Sheva 8410500, Israel; degen@bgu.ac.il

**Keywords:** yak, rumen-protected lysine, growth performance, blood metabolites, rumen fermentation, bacterial community

## Abstract

**Simple Summary:**

Yaks are the dominant livestock species on the Qinghai–Tibetan Plateau and provide the primary source of income for local pastoralists. Under traditional grazing management, production efficiency of alpine pastoral husbandry and feed efficiency are quite poor due to low forage intake during winter and the harsh environment. To improve production efficiency, a short-term intensive feedlot feeding program, based on a corn diet, has been introduced to finish yaks. However, corn is deficient in lysine; therefore, this study investigated the effect of rumen-protected lysine supplementation on growth performance, blood metabolites, rumen fermentation, and bacterial community on feedlot yaks offered corn-based diets. Rumen-protected lysine was associated with the improved performance of yaks fattened in feedlots on a corn-based diet.

**Abstract:**

Feedlots rely on corn-based total mixed rations (TMR) to finish yaks. However, corn is markedly deficient in lysine and, therefore, we hypothesized that feedlot yaks supplemented with rumen-protected lysine (RPLys) would improve performance. To test this hypothesis, twelve 2.5-year-old male yaks (122 ± 5.3 kg) were selected, and divided into a control (CON) and RPLys-supplemented (RPL) group. All yaks were provided with a pelleted diet that consisted of 25.0% corn stalk, 31.6% corn grain, and 24.0% corn by-products; while RPL yaks were supplemented with 37.0 g/d RPLys. Dry matter intake was not affected (*p* = 0.671) by RPLys supplementation, but the average daily gain was greater (*p* < 0.05; 1.46 vs. 1.25 kg/d) and the feed-to-gain ratio was lesser (*p* < 0.01; 3.39 vs. 3.90) in RPL than CON yaks. Serum urea nitrogen concentration and aspartate aminotransferase were greater (*p* < 0.05) in the CON than the RPL group. However, plasma lysine concentration was greater (*p* < 0.05), while threonine tended to be greater (*p* = 0.065) in RPL than CON yaks. Rumen ammonia-N concentration was lesser (*p* < 0.05) in RPL than CON yaks, but pH and volatile fatty acids concentration did not differ (*p* > 0.10) between groups. The relative abundances of the ruminal bacterial phyla of Firmicutes and Elusimicrobiota were greater (*p* < 0.05), whereas of the phylum Bacteroidota and genus *Butyrivibrio* were lesser (*p* < 0.05) in RPL than CON yaks. In general, the rumen microbiota was altered toward more abundant N utilization taxa in RPLys-supplemented yaks. RPLys-supplemented yaks had elevated plasma lysine and improved feed conversion ratio, providing the first evidence that bypass lysine improves the growth performance of yaks on corn-based diets in feedlots.

## 1. Introduction

Traditionally, yaks grazed the alpine rangeland without any supplements, a practice that failed to meet nutrient requirements of growing yaks, and thus constrained production efficiency [1]. Under this system, yaks gained only approximately 380 g/d overall [2], and were slaughtered at 5 years of age [3]. To improve production and shorten the time to slaughter, short-term intensive feedlot feeding has been introduced. This feeding regime increased the demand of feed resources, particularly of protein ingredients, since China imports more than 80% of its soybean meal, posing both economic and sustainability concerns [4,5]. Yaks raised in feedlots require more metabolizable protein (MP) than grazing yaks due to their faster growth rate, which led the farmers to greatly increase dietary protein levels in an attempt to maximize weight gain. However, ruminants offered high crude protein (CP) diets that exceed rumen microbial requirements not only increase feed costs, but also decrease N utilization efficiency [6,7]. Furthermore, excess CP increases urinary N and methane emission, causing environmental pollution [8,9]. The efficiency of MP utilization in ruminants depends on the profiles of the absorbable amino acids (AA) in the small intestine, since AA imbalances from the deficiency of a certain single AA could limit the utilization of other AA even when MP is sufficient [10].

To meet the MP requirements in ruminants, it is essential to consider both the amount of protein and the limiting AA (LAA) in the diet. Corn and corn products are used widely in ruminant diets, but then lysine (Lys) becomes the first LAA due to zein proteins [11,12,13]. Importantly, yaks require less nitrogen and utilize nitrogen more efficiently than cattle [14], and, therefore, Lys deficiency, rather than total protein, becomes the primary bottleneck in corn-based diets. Lys is one of the 20 AA used in the synthesis of body proteins and peptides [15], and diets deficient in Lys not only affect growth performance and carcass characteristics of livestock negatively, but also impair the immune function [15]. Lys added directly to the ruminant diet is degraded by Lys-utilizing bacteria through deamidation in the rumen [16], which subsequently reduces the total amount of AA available for absorption in the small intestine [17]. Bypassing the rumen would enable Lys to reach the small intestine [18,19], improve its utilization efficiency [20] and enhance ruminant performance [12,21,22].

RPLys supplementation has become a fundamental strategy for balancing AA nutrition in dairy cows [23,24], beef cattle [12], and sheep [25] with corn-based diets. However, the effects of RPLys supplementation on the blood AA profile, rumen fermentation, and feed conversion rate of feedlot yaks are still uncertain, as research on AA nutrition in yaks started relatively late. We hypothesized that rumen-protected lysine supplementation would improve growth performance and alter the bacterial community in feedlot-fattened yaks.

## 2. Materials and Methods

This study was conducted from July to September 2024, in a Farmers Cooperative in Hezuo County, located in the northeastern Qinghai–Tibetan Plateau.

### 2.1. Animal, Diets, and Experimental Design

Twelve 2-year-old male yaks (122 ± 5.3 kg) were selected from a herd, and divided randomly, stratified by bodyweight, into a control (CON, *n* = 6) group and a group supplemented with rumen-protected lysine (RPL, *n* = 6). The number of yaks was based on the effect size index (d-value), which was calculated using estimated standard deviations of the means of measured variables from previous similar studies. A sample size of six per group resulted in a d-value close to 0.5, which is a medium and acceptable effect size [26]. All yaks were provided with a pelleted basal total mixed ration (TMR) that consisted of 25.0% corn stalk, 31.6% corn grain, and 24.0% corn by-products; and the RPL yaks were supplemented with 37.0 g/d of rumen-protected lysine (RPLys). The RPLys was supplied by Hangzhou King Techina Feed Co., Ltd. (Hangzhou, China), and the lysine content was 60%. In an in situ experiment using rumen-cannulated Holstein cows, Lee et al. [27] reported that the rumen escape of RPLys was 89% and intestinal digestibility was 91%. The supplemented amount of lysine was calculated following Zinn et al. [28] and NRC [29], and was within the range recommended by the manufacturer of 14.2 g/d of metabolizable lysine.

The ingredients, chemical composition, and AA contents are presented in Table 1. The diet was formulated according to the Feeding Standard of Beef Cattle [30] with a concentrate-to-roughage ratio of 75:25. The crude protein and metabolizable energy of the diet were 12.1% and 10.6 MJ/kg DM, respectively.

The experiment lasted for 66 days, 10 days for acclimation to the conditions and 56 days for data collection. All yaks were tethered separately in the same shed and were fed daily at 08:30 and 18:00. The dry matter intake (DMI) of each yak was recorded daily by weighing the orts before morning feeding, and the orts were offered to non-experimental yaks. Fresh feed was offered so that approximately 10% remained to ensure that feed was ad libitum, and each yak had free access to clean water. RPLys was mixed with 100 g ground corn and top-dressed on the feed for RPL yaks before each morning feeding, whereas CON yaks were offered 100 g of ground corn. Both the RPLys and ground corn were consumed by the yaks within 2 min.

### 2.2. Sample Collection

The yaks were weighed before morning feeding at the beginning and end of the experiment, and the initial body weight (IBW) and final body weight (FBW) were used to calculate the average daily gain (ADG). On days 1, 28, and 56, approximately 300 g of the diet were collected and frozen at −20 °C. On day 57, before morning feeding, 15 mL of jugular vein blood were collected from each yak using vacutainers: 5 mL were placed in heparinized tubes and 10 mL in non-heparinized tubes. The heparinized blood was centrifuged at 2000× *g* for 6 min while the non-heparinized blood was kept on ice for 30 min and centrifuged at 3500× *g* for 10 min. Both the plasma and serum were stored in 1.5 mL tubes at −20 °C. In addition, approximately 150 mL of rumen fluid were collected from each yak using an oral stomach tube (Anscitech Co., Ltd., Wuhan, China) attached to a vacuum pump. The tube was washed thoroughly between collections, and the first 50 mL were discarded to minimize saliva contamination. Ruminal pH was measured immediately using a portable pH meter (PHB-4, INESA Analytical Instrument Co., Ltd., Shanghai, China), and then the rumen fluid was filtered through four layers of cheesecloth. Five mL aliquots were processed as follows: one portion was mixed with an equal volume of deproteinizing solution (100 g metaphosphoric acid plus 0.6 g crotonic acid per liter) for measurement of volatile fatty acids (VFA); a second portion was mixed with 0.5 mmol/L HCl for ammonia determination; a third portion was stored for rumen bacterial 16S rDNA sequencing; all samples were stored at −20 °C.

### 2.3. Laboratory Analyses

Feed samples were dried at 65 °C for 48 h, air-equilibrated, ground to pass through a 1 mm screen, and stored in sealed plastic bags. Following AOAC [32], dry matter (DM) content was determined by oven-drying at 105 °C for 4 h (method 934.01); nitrogen was measured by the Kjeldahl method (method 954.01), and crude protein was calculated as N × 6.25; ether extract (EE) was determined by a reflux system using petroleum ether extracted at 90 °C for 1 h (method 920.39); and crude ash was determined by complete combustion in a muffle furnace at 600 °C for 6 h (method 938.08). Neutral detergent fiber (NDF) and acid detergent fiber (ADF), including residual ash, were measured by an automatic fiber analyzer (Ankom Technology, Fairport, NY, USA) following the methods of Robertson and Van Soest [33] and Van Soest et al. [34], respectively. Feed and plasma AA profiles were analyzed by HPLC (LC-20A, Shimadzu, Kyoto, Japan) using a column of 150 mm × 4.6 mm × 5 µm (Venusil MP C18, Agela, Tianjin, China), following Li et al. [35].

Serum total protein (TP), albumin (ALB), globulin (GLO), blood urea nitrogen (BUN), creatinine (CRE), glucose (GLU), alanine aminotransferase (ALT), aspartate aminotransferase (AST), total glyceride (TG), and cholesterol (CHO) were determined using commercial kits and an automatic biochemistry analyzer (Hitachi 7160, Hitachi High-Technologies Corporation, Tokyo, Japan), following the manufacturer’s instructions (Hunan Fengrui Biotechnology Co., Ltd., Changsha, China).

Ruminal volatile fatty acids (VFA) concentrations were analyzed by gas chromatography (Trace 1300, Thermo Scientific, Waltham, MA, USA) equipped with a capillary column (AT-FFAP, 15 m × 0.32 mm × 0.5 μm), following Liu et al. [22]. Ruminal ammonia-N concentration was determined by colorimetry method [36] using a spectrometer (SpectraMax M5, Molecular Devices, San Jose, CA, USA).

Total microbial DNA was extracted from thawed rumen fluid samples (*n* = 6 per treatment) using the E.Z.N.A. DNA Kit (Omega Bio-tek, Norcross, GA, USA), following the manufacturer’s instructions. The purity and concentration of extracted DNA were verified using a spectrophotometer (NanoDrop 2000UV-vis, Thermo Scientific, Wilmington, DE, USA), with a purity ratio (260/280 nm) ranging from 1.8 to 2.0. The quality of DNA was examined using 1% agarose gel electrophoresis. The V3 and V4 highly variable regions of the rumen bacterial 16S rRNA gene were amplified by a PCR amplifier (GeneAmp 9700, ABI, Foster City, CA, USA) using the universal forward primer 338F (5′-ACTCCTACGGGAGGCAGCAG-3′) and the reverse primer 806R (5′-GGACTACHVGGGTWTCTAAT-3′), following PCR reaction procedures as described by Wei et al. [37]. The PCR product was extracted from 2% agarose gels, purified using the AxyPrep DNA Gel Extraction Kit (Axygen Biosciences, Union City, CA, USA), and quantified using a Quantus™ Fluorometer (Promega, Madison, WI, USA). The amplicons were sequenced on the Illumina MiSeq PE300 platform (Illumina, San Diego, CA, USA).

Raw FASTQ files were quality filtered by Trimmomatic and merged by FLASH with the following criteria: (1) the reads were truncated at any site receiving an average quality score below 20 over a 50 bp sliding window; (2) sequences with an overlap length of more than 10 bp were merged, with mismatch error rates of 2%; and (3) individual samples were separated based on barcodes and primers.

### 2.4. Statistical Analyses

Growth performance, serum biochemical parameters, plasma AA profiles, rumen fermentation variables, and α-diversity indices were compared between the CON and RPL groups using an independent samples *t*-test (SPSS 22 version, IBM Corp., Chicago, IL, USA). The results are presented as means and standard error of the mean (SEM). Difference between means was accepted as significant at *p* ≤ 0.05 and as a tendency to differ at 0.05 < *p* ≤ 0.10. Rumen bacterial composition was compared between yak groups using the non-parametric Wilcoxon rank-sum test, with the FDR correction of multiple tests. The plots were generated using the online platform (https://www.majorbio.com/, accessed on 23 March 2025).

## 3. Results

### 3.1. Growth Performance and Feed Conversion Efficiency

Dry matter intake (DMI) ranged between 4.85 and 4.91 kg/d and did not differ (*p* = 0.671) between yak groups (Table 2). There was no difference (*p* > 0.10) in IBW or FBW between CON and RPL yaks; however, ADG was greater (*p* < 0.05; 1.46 vs. 1.25 kg/d) and the feed to gain ratio (FGR) was lesser (*p* < 0.01; 3.39 vs. 3.90) in RPL than CON yaks (Table 2).

### 3.2. Serum Biochemical

Serum BUN concentration was lesser (*p* < 0.05) in RPL than CON yaks, while TP, ALB, and GLO did not differ (*p* > 0.10) between groups. Serum concentration of ALT was greater (*p* < 0.05) in CON than RPL yaks, while AST did not differ (*p* = 0.867) between groups. In addition, serum concentrations of CRE, GLU, TG, and CHO did not differ (*p* > 0.10) between groups (Table 3).

### 3.3. Plasma Amino Acid Profiles

Plasma Lys concentration was greater (*p* < 0.05), and threonine concentration tended to be greater (*p* = 0.065) in RPL than CON yaks. However, concentrations of the other AA, total essential amino acids (EAA), total non-essential amino acids (NEAA), and total AA did not differ (*p* > 0.10) between yak groups (Table 4).

### 3.4. Rumen Fermentation Parameters

Ruminal pH and total VFA concentration did not differ (*p* > 0.10) between groups, whereas ammonia-N concentration was lesser (*p* < 0.01) in RPL than CON yaks. In addition, the molar proportion of the individual VFA or the acetate:propionate ratio in the rumen did not differ (*p* > 0.10) between groups (Table 5).

### 3.5. Bacterial Community Composition

**Figure 1:**
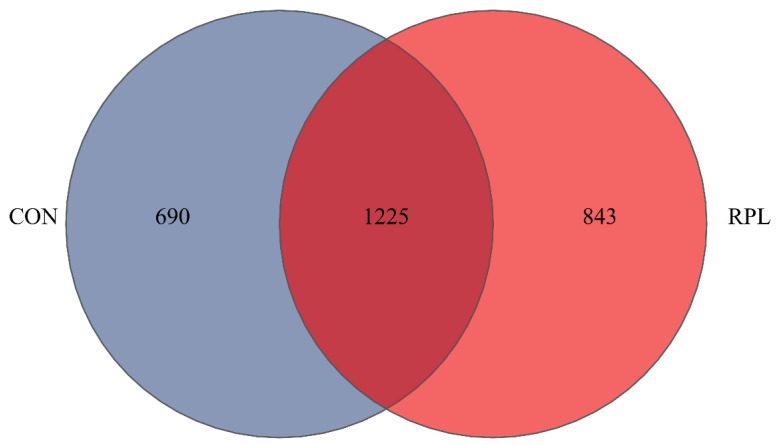
Venn plot illustrating the different and similar OTUs between CON and RPL yaks.

A total of 927,362 raw reads were generated from the 12 rumen fluid samples through the 16S rRNA sequencing, and 915,596 high-quality reads were selected after data processing, with an average sequence length of 420 bp. In total, 2758 operational taxonomic units (OTUs) were obtained based on 97% similarity level according to nucleotide sequence identification. There were 1225 OTUs shared between these two groups, and the specific OTUs in CON and RPL yaks were 690 and 843, respectively (Figure 1).

For the alpha diversities of the rumen bacteria, the Shannon index tended to be greater (*p* = 0.077) in RPL than CON yaks, whereas the Chao 1 and ACE indices did not differ (*p* > 0.10) between groups (Table 6).

**Figure 2:**
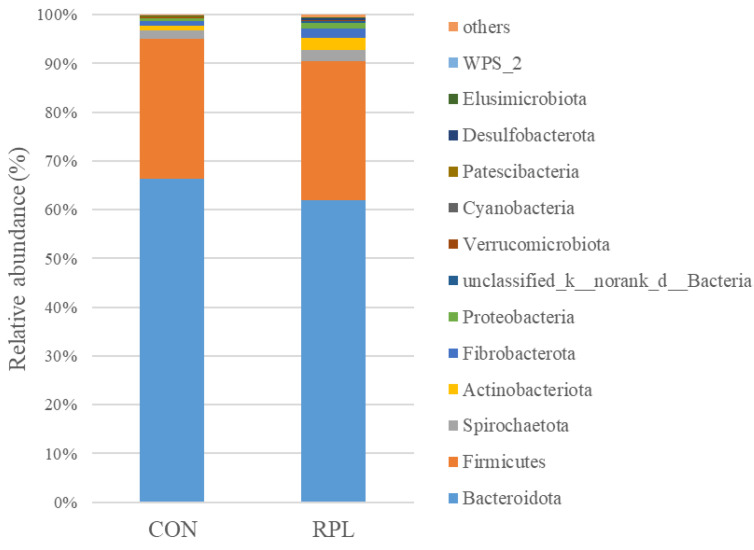
Effect of supplementary rumen-protected lysine on rumen bacterial community at the phylum level (abundance > 0.05%) in fattening feedlot yaks.

A total of 19 rumen bacterial phyla were identified in the yaks, of which 13 phyla had a relative abundance above 0.05% (Figure 2). The dominant phylum was Bacteroidota, with relative abundances of 66.4% and 62.3% for CON and RPL yaks, respectively; followed by Firmicutes with relative abundances of 28.9% and 28.7% for CON and RPL yaks, respectively. The relative abundance of Elusimicrobiota was greater (*p* < 0.05) in the RPL than CON yaks, while the remaining bacterial phyla did not differ between groups.

**Figure 3:**
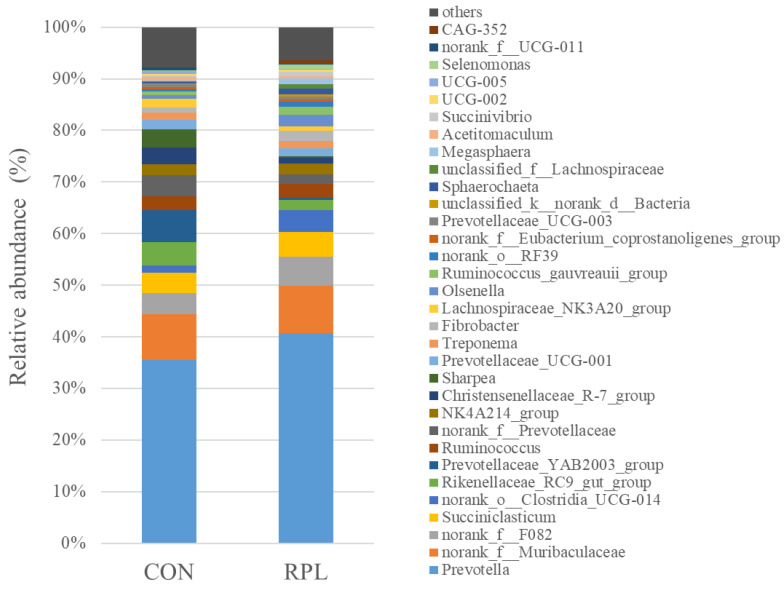
Effect of supplementary rumen-protected lysine on rumen bacterial community at the genus level (abundance > 0.05%) in fattening feedlot yaks.

A total of 301 bacterial genera was identified, and 32 genera had a relative abundance greater than 0.5% (Figure 3). The most abundant genus was *Prevotella* (37.5%), followed by *noran_f_Muribaculaceae* (8.92%), and then *norank_f_F082* (4.76%). The relative abundances of *Elusimicrobium*, *Eubacterium_xyanophilum_group*, *norank_c_Clostridia*, and *lachnospiraceae_UCG-001* were greater (*p* < 0.05), whereas of *Butyrivibrio*, *Eubacterium_brachy_group*, and *Romboutsia* were lesser (*p* < 0.05) in RPL than CON yaks.

**Figure 4:**
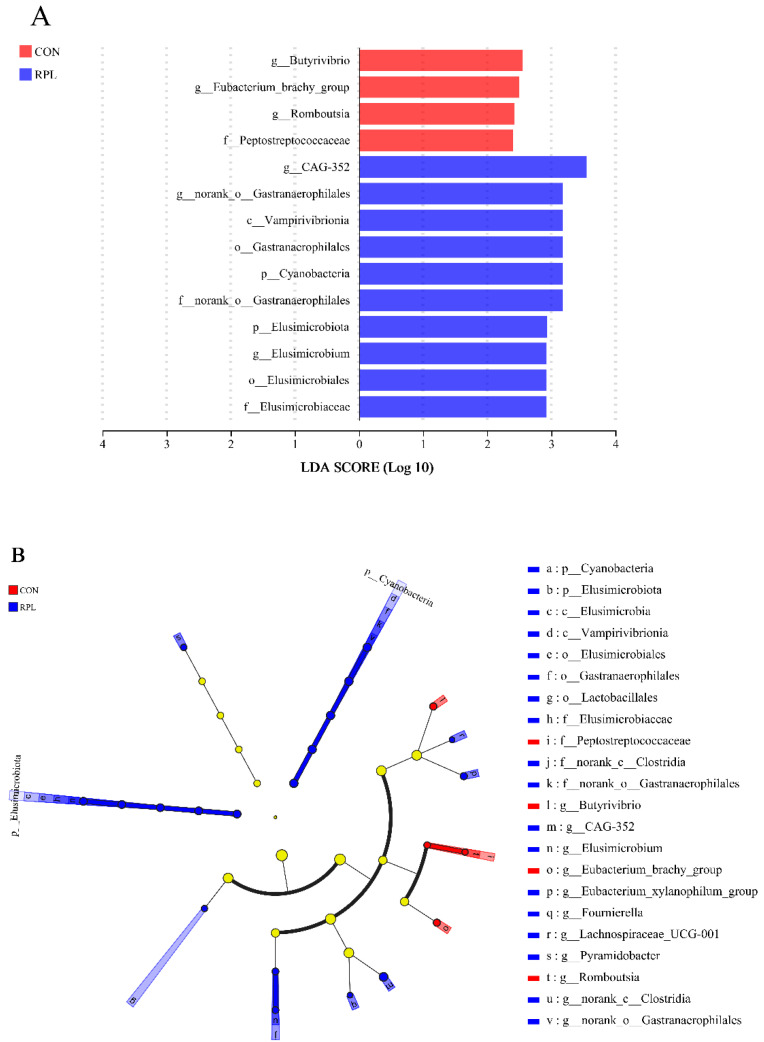
Linear discriminant analysis effect size (LEfSe) for rumen bacterial community. (**A**) LEfSe analysis LDA histograms (LDA score > 2.0); (**B**) LEfSe analysis cladogram of the characteristic microorganisms. Prefixes represent taxonomic ranks: phylum, p; class, c; order, o; family, f and genus, g.

Differential rumen bacteria distinguished between CON and RPL yaks were further identified by linear discriminant analysis effect size (LEfSe; Figure 4). With a default LDA cutoff of ±4.0, the differential taxa for the CON and RPL yaks totaled 4 and 10 genera, respectively. *Butyrivibrio*, *Eubacterium_brachy*, and *Romboutsia*, genera in the CON yaks, and *CAG_352*, *norank_o_Gastranaerophilales*, and *Elusimicrobium*, genera in the RPL yaks, had high impacts on the difference between treatments.

## 4. Discussion

### 4.1. Effect of Supplementary Rumen-Protected Lysine on Growth Performance and Feed Conversion in Fattening Yaks

Traditionally, yaks grazed alpine rangeland all year round without supplements. The yaks would lose approximately 25% of their liveweight during the cold season [38], while the ADG was only approximately 400 g/d in the warm season [39,40]. As a result, the production of grazing yaks was low. In agreement with previous studies in ruminants [21,41], supplementation with RPLys in the present study did not affect DMI. However, the ADG of CON and RPL yaks were 1.25 and 1.46 kg/d, respectively, which were approximately three times the ADG of grazing yaks. The ADG was greater by 16.8% in the RPL than CON yaks and, therefore, the feed conversion rate was enhanced in yaks supplemented with RPLys. Consequently, the short-term intensive fattening strategy with RPLys provides an economically attractive option for yak producers. Similarly, a quadratic effect on ADG was reported in finishing calves when offered a corn-based diet supplemented with increasing dosages of RPLys (providing metabolizable lysine levels of 0, 1, 2, 3, 4, 6, 8, 10, or 12 g/d). The calves supplemented with 3 g/d had an ADG that was approximately 15% greater than the non-dosed calves [12].

In previous studies, the feed conversion ratios for fattening yaks and beef cattle fed highly concentrated feed ranged from 6.41 to 8.16 [42,43] and from 9.09 to 13.8 [42,44], respectively, while it ranged between 3.39 and 3.90 in the present study. The remarkably low feed conversion rate in the present study was probably due to compensatory growth [38,45]. The yaks in the present study grazed only on pasture prior to this study, and we reasoned that the compensatory growth occurred when switched to stall-feeding with a high-energy diet. Likewise, compensatory growth was reported in grazing yaks when pasture was shifted from withered to green grass [2]. The dietary CP content in the present study was 12.1%, whereas CP content in previous yak studies in feedlots typically ranged between 16 and 18% [46,47]. However, the ADG in the present study was considerably greater than that of yaks offered high CP diets (1.3 kg/d versus 0.8 kg/d). Yaks are known for their “N-saving” characteristics [48,49], and for their low nitrogen requirement for maintenance [14]. A reduction in dietary CP would reduce feed costs without decreasing growth performance of finishing yaks in feedlots.

### 4.2. Effect of Supplementary Rumen-Protected Lysine on Serum Biochemical Parameters in Fattening Feedlot Yaks

Blood biochemical variables are important indicators of the nutritional metabolism and health status in ruminants [50]. Urea is a metabolic end product of AA and, therefore, BUN reflects the status of N absorption and metabolism [51], and is correlated negatively with N utilization efficiency [52]. In the present study, serum BUN concentration was lesser in RPL than CON yaks, indicating an increase in protein synthesis and that nitrogen utilization was more efficient in RPL yaks, which could explain, at least in part, the greater ADG in the RPL than CON group [53]. Similarly, it was reported that plasma BUN concentration decreased when dairy cows were offered a low CP diet supplemented with 40 g/d RPLys [37]. The reasons probably were as follows: (1) RPLys supplementation improved the AA balance in the small intestine, allocating more N for body tissues [20], subsequently reducing AA oxidation [54] and N waste [53,55]; and (2) some supplemented RPLys was degraded in the rumen, which promoted the utilization of ammonia by rumen microorganisms and improved the synthesis efficiency of microbial proteins (MCP) [37]. The decrease in rumen ammonia concentration would reduce excess ammonia transfer to the liver and reduce urea synthesis through the ornithine cycle [56].

Serum TP consists mainly of ALB and GLO, and previous studies reported that ADG was correlated positively with serum TP concentration. In the present study, TP, ALB and GLO did not differ between groups, but all were within the normal range of ruminants [50,57]. Socha et al. [58] reported that RPLys supplementation increased plasma GLU concentrations in dairy cows. However, this effect was not observed in the present study, which was perhaps associated with the similar DMI, absolute rumen total VFA concentration, and molar proportion of propionate between CON and RPL yaks. Both yak groups were well below the renal threshold for GLU of 5.56 to 7.78 mmol/L for cattle [59]. ALT is an enzyme that exists predominantly in the liver, participates in AA metabolism and GLU synthesis, and is considered as a biomarker for evaluation of liver injury [60]. In the present study, serum ALT activity was greater in CON than RPL yaks, but both yak groups were within the normal range (6.90–35.3 U/L) [61], indicating normal liver function. Perhaps RPLys supplementation could reduce the risk of liver injury.

### 4.3. Effect of Supplementary Rumen-Protected Lysine on Plasma-Free Amino Acid Profiles in Fattening Yaks

Plasma-free AA reflects the sum of metabolic AA from all organs and tissues [62] and can be used to evaluate and compare AA availability [63]. The increase in plasma Lys concentration in the RPL treatment was attributed to dietary RPLys supplementation, which increased duodenal Lys flow [55], resulting in an increase in Lys available in the small intestine [20] and enabling more Lys to enter the circulation. This reasoning was supported in studies in dairy cows [64] and growing cattle [21] in which plasma Lys concentration increased with an increase in metabolizable Lys in the small intestine. However, in other studies in ruminants, plasma Lys concentration was not affected by PRLys supplementation [23,24]. This discrepancy among studies could be due to differences in dosages of RPLys, form of RPLys, animal species, and physiological status of the animals [19]. Xue et al. [21] reported that plasma threonine concentration in crossbred bulls decreased linearly with increasing dosages of RPLys supplementation; however, plasma threonine concentration of RPL yaks tended to increase with RPLys supplementation in the present study. The changes in plasma threonine with RPLys supplementation were probably induced by specific synergistic or antagonistic effects between lysine and threonine in the small intestine, the mechanism of which warrants further investigation. In general, if essential AA exceed the nutritional requirements of the animal, the rate of catabolism and transamination of Lys would increase, and then produce NEAA [65]. In the present study, there was no difference in plasma NEAA concentration between CON and RPL yaks, which indicated that RPLys supplementation was not in excess.

### 4.4. Effect of Supplementary Rumen-Protected Lysine on Rumen Fermentation Parameters in Fattening Yaks

Ruminal fermentation parameters are important indicators of the physiological status of the animal. Typically, rumen pH is correlated negatively with total VFA concentration [66]. In the present study, RPLys supplementation did not affect either rumen pH or total VFA concentration. The rumen pH of the yak groups ranged between 7.10 and 7.15, which fell within the optimal range of 6.2–7.2 for microbial function [67]. Ammonia-N is an end product of dietary proteins, peptides, AA, and non-protein N in the rumen, and is considered to be the most important nitrogen source for MCP synthesis. Rumen ammonia-N concentration reflects the homeostasis between ammonia generation for microbial catabolism of dietary proteins and ammonia utilization for MCP synthesis. An appropriate ammonia-N concentration is important for rumen fermentation and MCP synthesis, since a low concentration inhibits MCP synthesis, whereas an excessively high concentration could cause ammonia toxicity [68]. In the present study, RPL yaks had a lesser rumen ammonia-N concentration, which was consistent with a previous study in goats supplemented with lysine [55], but rumen ammonia-N concentrations in both CON and RPL yaks were within the optimal range of 2.8–17.8 mmol/L [69]. The lesser rumen ammonia concentration in the RPL yaks suggested that the portion of bypass lysine released in the rumen improved MCP synthesis, accompanied by an increase in ammonia utilization by rumen microorganisms, and reduced the ruminal ammonia pool [37,70]. The decrease in serum BUN concentration, along with reports by Tu et al. [71] who reported that supplementation with 0.15% and 0.30% RPLys increased the apparent digestibility of CP in yaks, further supported this inference. In a previous study in lactating dairy cows, the addition of 125 g/d RPlys had no effect on ruminal total VFA concentration or individual molar proportions [23], which was in agreement with the results of present study. The yak groups were offered the same basal diets and there was no difference in DMI.

### 4.5. Effect of Supplementary Rumen-Protected Lysine on Rumen Bacterial Diversity and Bacterial Community Composition

The α-diversity indices reflect the abundance and diversity of bacterial species in the rumen [72]. In line with previous findings [53,73], the bacterial α-diversities of Chao 1 and ACE were not affected by RPLys supplementation. However, the Shannon diversity index tended to be greater in RPL than CON yaks, which indicated a greater bacterial community diversity in the RPL than CON yaks [74]. Dietary ingredients and nutritional composition are the primary factors influencing rumen microbial community, fermentable function, and metabolic end products [75]. The CON and RPL yaks were offered the same basal diets, and the DMI and yak physiological conditions were comparable, with the only difference being the intake of RPLys in the RPL yaks. This would suggest that the difference in the Shannon diversity index was due to the RPLys.

In agreement with previous studies in ruminants, Bacteroidota and Firmicutes were the dominant bacterial phyla in the rumen [50,76], and they are involved mainly in carbohydrate and protein metabolism [73]. It was reported that the ratio of Firmicutes and Bacteroidota increased with dietary energy level and correlated positively with energy conversion efficiency and fat deposition [76]. The numerically greater Firmicutes:Bacteroidota ratio in RPL than CON yaks could be related to the greater ADG observed in the RPL than CON yaks. Elusimicrobiota is distributed in the gastrointestinal tracts of monogastric and ruminant animals [77,78], and also exists in sediments, soils, and groundwater [79]. This bacterial phylum could fix N_2_ by synthesizing and secreting the N-fixing enzyme Group IV [80]. The RPL yaks had a greater relative abundance of Elusimicrobiota but lesser relative abundance of *Butyrivibro*. Alves et al. [81] reported that Elusimicrobiota was correlated negatively with urinary N excretion, while *Butyrivibrio* was correlated positively with urinary N excretion and negatively with N retention. Therefore, it could be inferred that RPLys supplementation improved N utilization by increasing the relative abundance of Elusimicrobiota and decreasing the relative abundance of *Butyrivibrio*. Previous studies reported that AA supplementation increased the growth of rumen cellulolytic bacteria and promoted fiber digestion in lactating dairy cows [82], while RPLys supplementation increased the apparent digestibility of NDF in yaks [71]. This could explain why the abundances of cellulose degraders [83,84], such as *g_Eubacterium_xylanophilum_group* and *Lachnospiraceae_UCG-001*, were greater in RPL than CON yaks.

## 5. Conclusions

The ADG was greater and feed-to-gain ratio was lesser in RPL than CON yaks; however, DMI and rumen VFA profiles did not differ between groups. Serum BUN and rumen ammonia-N concentrations were lesser, while plasma Lys concentration was greater in RPL than CON yaks. RPLys supplementation increased abundances of Firmicutes and Elusimicrobiota and decreased abundances of Bacteroidota and *Butyrivibrio*. RPLys was associated with an improved performance in yaks fattened in feedlots. The present study is the first to link RPLys with shifts in the yak microbiome, but more studies are warranted, including dose–response trials and carcass quality measurements, to determine the optimal dose for yaks.

## Figures and Tables

**Table 1:** Ingredients, chemical composition, and amino acids of the pellets offered to the yaks.

Items	Content	Items	Content
Ingredients (%, air-dried basis)		AA composition (μg/mg, DM basis)	
Corn stalk	25.0	Alanine	0.361
Corn grain	31.6	Asparagine	0.402
Sprayed corn bran	15.0	Aspartic acid	0.235
Corn germ meal	6.00	Glutamine	0.099
Distillers dried grains with soluble	3.00	Glutamic acid	0.287
Soybean meal	4.50	Glycine	0.055
Cottonseed meal	2.00	Histidine	0.084
Molasses	3.00	Proline	0.349
Wheat bran	6.00	Serine	0.157
Soybean oil	0.50		
CaCO_3_	1.40		
NaCl	0.50		
NaHCO_3_	0.50		
Premix ^1^	1.00		
Chemical composition (%, DM basis)			
DM	89.9		
CP	12.1		
EE	2.73		
NDF	33.2		
ADF	14.4		
Ash	5.85		
ME, MJ/kg ^2^	10.6		

AA, amino acid; ADF, acid detergent fiber; Ash, crude ash; CP, crude protein; DM, dry matter; EE, ether extract; ME, metabolizable energy; NDF, neutral detergent fiber. ^1^ The premix provided the following per kg: vitamin A, 3,000,000 IU; vitamin D, 375,000 IU; vitamin E, 220 IU; Fe, 3000 mg; Mn, 2000 mg; Zn, 4000 mg; Cu, 1200 mg; I, 15 mg; Se, 20 mg; Co, 16 mg. ^2^ The ME was calculated according to the Tables of Feed Composition and Nutritive Values in China (33rd edition, 2022) [31], while the rest are measured values.

**Table 2:** Effect of supplementary rumen-protected lysine on growth performance and feed conversion efficiency in fattening feedlot yaks.

Items	Treatment		SEM	*p*-Value
	CON	RPL		
IBW, kg	121	123	5.3	0.861
FBW, kg	191	205	6.5	0.319
ADG, kg/d	1.25	1.46	0.053	0.043
DMI, kg/d	4.85	4.91	0.063	0.671
Feed conversion ratio ^1^	3.90	3.39	0.108	<0.01

ADG, average daily gain; CON, control; DMI, dry matter intake; FBW, final body weight; IBW, initial body weight; RPL, rumen-protected lysine; SEM, standard error of the means. ^1^ Feed conversion ratio = DMI:ADG.

**Table 3:** Effect of supplementary rumen-protected lysine on serum biochemical parameters in fattening feedlot yaks.

Items	Treatment		SEM	*p*-Value
	CON	RPL		
TP, g/L	60.9	60.1	1.10	0.761
ALB, g/L	35.4	35.0	0.59	0.781
GLO, g/L	25.5	25.1	0.95	0.857
BUN, mmol/L	5.70	4.77	0.230	0.036
CRE, μmol/L	34.3	34.3	0.76	0.984
GLU, mmol/L	4.64	4.80	0.095	0.414
ALT, U/L	27.2	20.2	1.81	0.047
AST, U/L	80.2	83.3	8.77	0.867
TG, mmol/L	0.202	0.155	0.018	0.211
CHO, mmol/L	1.45	1.55	0.087	0.591

ALB, albumin; ALT, alanine aminotransferase; AST, aspartate aminotransferase; BUN, blood urea nitrogen; CHO, cholesterol; CON, control; CRE, creatinine; GLO, globulin; GLU, glucose; RPL, rumen-protected lysine; SEM, standard error of the means; TG, total glyceride; TP, total protein.

**Table 4:** Effect of supplementary rumen-protected lysine on plasma-free amino acid profiles in fattening feedlot yaks.

Items	Treatment		SEM	*p*-Value
	CON	RPL		
EAA, mg/L				
Arginine	15.0	13.2	1.03	0.400
Histidine	10.0	10.1	0.56	0.922
Isoleucine	11.5	11.0	0.80	0.750
Leucine	15.1	18.0	1.05	0.179
Lysine	10.9	14.3	0.81	0.028
Methionine	2.53	3.04	0.253	0.344
Phenylalanine	8.00	9.10	0.647	0.421
Threonine	5.39	8.06	0.734	0.065
Tryptophan	6.01	5.97	0.493	0.972
Valine	27.0	27.0	2.13	0.994
Total EAA	111	120	6.8	0.565
NEAA, mg/L				
Alanine	30.4	27.6	1.50	0.381
Asparagine	4.88	4.91	0.466	0.972
Aspartic acid	0.765	0.552	0.140	0.472
Cysteine	4.15	4.66	0.432	0.581
Glutamine	36.7	33.8	1.42	0.335
Glutamic acid	6.15	6.22	0.440	0.937
Glycine	22.5	19.0	2.24	0.474
Proline	10.9	9.72	0.393	0.134
Serine	9.72	7.59	0.736	0.156
Tyrosine	8.67	9.02	0.987	0.870
Total NEAA	135	123	5.8	0.337
Total AA	246	243	11.8	0.897

CON, control; EAA, essential amino acids; NEAA, non-essential amino acids; RPL, rumen-protected lysine; SEM, standard error of the means; total AA, total amino acids.

**Table 5:** Effect of supplementary rumen-protected lysine on rumen fermentation parameters in fattening feedlot yaks.

Items	Treatment		SEM	*p*-Value
	CON	RPL		
pH	7.15	7.10	0.132	0.881
Ammonia-N, mmol/L	5.32	2.99	0.488	<0.01
Total VFA, mmol/L	64.1	61.5	3.92	0.764
Individual VFA, mol/100 mol				
Acetate	62.0	61.2	1.43	0.794
Propionate	24.1	26.5	1.65	0.514
Isobutyrate	1.14	1.09	0.060	0.722
Butyrate	10.0	8.36	0.626	0.204
Isovalerate	1.39	1.37	0.106	0.934
Valerate	1.35	1.56	0.145	0.500
Acetate: Propionate ratio	2.75	2.39	0.242	0.483

CON, control group; N, nitrogen; RPL, rumen-protected lysine; SEM, standard error of the means; VFA, volatile fatty acid.

**Table 6:** Effect of supplementary rumen-protected lysine on alpha diversity of the rumen bacterial community in fattening feedlot yaks.

Items	Treatment		SEM	*p*-Value
	CON	RPL		
Chao 1	737	840	35.8	0.160
ACE	761	864	34.4	0.140
Shannon	3.58	4.22	0.183	0.077

CON, control group; RPL, rumen-protected lysine; SEM, standard error of the means.

## Data Availability

The original data in this study are included in the manuscript. Further inquiries can be directed to the corresponding author.

## References

[1] Zhang Q.Y., Jiao J.X., Zhao Z.W., Ma Z.Y., Kakade A., Jing X.P., Mi J.D., Long R.J. (2025). Feeding systems change yak meat quality and flavor in cold season. Food Res. Int..

[2] Ding L.M., Chen J.Q., Long R.J., Gibb M.J., Wang L., Sang C., Mi J.D., Zhou J.W., Liu P.P., Shang Z.H. (2015). Blood hormonal and metabolite levels in grazing yak steers undergoing compensatory growth. Anim. Feed Sci. Technol..

[3] Bai X.Y., Yin F., Ru A., Tian W., Chen Q.W., Chai R., Liu Y.X., Cui W.M., Li J.H., Yin M.C. (2023). Effect of slaughter age and postmortem aging time on tenderness and water-holding capacity of yak (*Bos grunniens*) longissimus thoracis muscle. Meat Sci..

[4] Yin J., Liu H.N., Li T.J., Yin Y.L. (2019). Current situation and developmental suggestions on shortage of feeding protein resources in Chinese pig industry. Bull. Chin. Acad. Sci..

[5] MARA (2022). China Animal Husbandry and Veterinary Yearbook.

[6] Broderick G.A. (2003). Effects of varying dietary protein and energy levels on the production of lactating dairy cows. J. Dairy Sci..

[7] Danes M.A.C., Chagas L.J., Pedroso A.M., Santos F.A.P. (2013). Effect of protein supplementation on milk production and metabolism of dairy cows grazing tropical grass. J. Dairy Sci..

[8] Olmos Colmenero J.J., Broderick G.A. (2006). Effect of dietary crude protein concentration on milk production and nitrogen utilization in lactating dairy cows. J. Dairy Sci..

[9] Abbasi I.H.R., Abbasi F., Abd El-Hack M.E., Abdel-Latif M.A., Soomro R.N., Hayat K., Mohamed M.A.E., Bodinga B.M., Yao J.H., Cao Y.C. (2018). Critical analysis of excessive utilization of crude protein in ruminants ration: Impact on environmental ecosystem and opportunities of supplementation of limiting amino acids—A review. Environ. Sci. Pollut. Res..

[10] Cole D.J.A., Van Lunen T.A., D’ Mello J.F.P. (1994). Ideal Amino Acid Patterns. Amino Acids in Farm Animal Nutrition.

[11] Williams J.E., Newell S.A., Hess B.W., Scholljegerdes E. (1999). Influence of rumen-protected methionine and lysine on growing cattle fed forage and corn based diets. J. Prod. Agric..

[12] Klemesrud M.J., Klopfenstein T.J., Stock R.A., Lewis A.J., Herold D.W. (2000). Effect of dietary concentration of metabolizable lysine on finishing cattle performance. J. Anim. Sci..

[13] Weiss W.P. (2019). Effects of feeding diets composed of corn silage and a corn milling product with and without supplemental lysine and methionine to dairy cows. J. Dairy Sci..

[14] Zhou J.W., Zhong C.L., Liu H., Degen A.A., Titgemeyer E.C., Ding L.M., Shang Z.H., Guo X.S., Qiu Q., Li Z.P. (2017). Comparison of nitrogen utilization and urea kinetics between yaks (*Bos grunniens*) and indigenous cattle (*Bos taurus*). J. Anim. Sci..

[15] Liao S.F., Wang T.J., Regmi N. (2015). Lysine nutrition in swine and the related monogastric animals: Muscle protein biosynthesis and beyond. SpringerPlus.

[16] Garner M.R., Flint J.F., Russell J.B. (2002). *Allisonella histaminiformans* gen.nov., sp. nov. A novel bacterium that produces histamine, utilizes histidine as its sole energy source, and could play a role in bovine and equine laminitis. Syst. Appl. Microbiol..

[17] Robinson P.H., DePeters E.J., Shinzato I., Sato H. (2006). Rumen lysine escape, rumen fermentation, and productivity of early lactation dairy cows fed free lysine. Anim. Feed Sci. Technol..

[18] Mazinani M., Erdogan M., Brian J.R. (2022). Harnessing the value of rumen protected amino acids to enhance animal performance—A review. Ann. Anim. Sci..

[19] Kaur J., Kaur R., Mahesh M.S., Thakur S.S. (2024). Rumen-protected amino acids for ruminants. Feed Additives and Supplements for Ruminants.

[20] Fleming A.J., Lapierre H., White R.R., Tran H., Kononoff P.J., Martineau R., Weiss W.P., Hanigan M.D. (2019). Predictions of ruminal outflow of essential amino acids in dairy cattle. J. Dairy Sci..

[21] Xue F., Zhou Z.M., Ren L.P., Meng Q.X. (2011). Influence of rumen-protected lysine supplementation on growth performance and plasma amino acid concentrations in growing cattle offered the maize stalk silage/maize grain-based diet. Anim. Feed Sci. Technol..

[22] Liu H., Yang G., Degen A., Ji K.X., Jiao D., Liang Y.P., Xiao L., Long R.J., Zhou J.W. (2021). Effect of feed level and supplementary rumen protected lysine and methionine on growth performance, rumen fermentation, blood metabolites and nitrogen balance in growing Tan lambs fed low protein diets. Anim. Feed Sci. Technol..

[23] Lobos N.E., Wattiaux M.A., Broderick G.A. (2021). Effect of rumen-protected lysine supplementation of diets based on corn protein fed to lactating dairy cows. J. Dairy Sci..

[24] Malacco V.M.R., Beckett L., Hilger S., Doane P., Reis R.B., Donkin S.S. (2022). Effects of increased doses of lysine in a rumen-protected form on plasma amino acid concentration and lactational performance of dairy cows fed a lysine-deficient diet. J. Dairy Sci..

[25] Han I.K., Ha J.K., Lee S.S., Ko Y.G., Lee H.S. (1996). Effect of supplementing rumen-protected lysine on growth performance and plasma amino acid concentrations in sheep. Asian-Australas. J. Anim. Sci..

[26] Sullivan G.M., Feinn R. (2012). Using effect size—Or why the *P* value is not enough. J. Grad. Med..

[27] Lee C., Hristov A.N., Cassidy T.W., Heyler K.S., Lapierre H., Varga G.A., de Veth M.J., Patton R.A., Parys C. (2012). Rumen-protected lysine, methionine, and histidine increase milk protein yield in dairy cows fed a metabolizable protein-deficient diet. J. Dairy Sci..

[28] Zinn R.A., Shen Y. (1988). An evaluation of ruminally degradable intake protein and metabolizable amino acid requirements of feedlot calves. J. Anim. Sci..

[29] NRC (2016). Nutrient Requirements of Beef Cattle.

[30] (2004). Feeding Standard of Beef Cattle.

[31] Xiong B.H., Luo Q.R., Zheng S.S., Zhao Y.G. (2022). Tables of Feed Composition and Nutritive Values in China, 33rd ed. China Feed.

[32] AOAC (2016). Official Methods of Analysis.

[33] Robertson J.B., Van Soest P.J. (1981). The Detergent System of Analysis and its Application to Human Foods. The Analysis of Dietary Fibre in Food.

[34] Van Soest P.J., Robertson J.B., Lewis B.A. (1991). Methods for dietary fiber, neutral detergent fiber, and nonstarch polysaccharides in relation to animal nutrition. J. Dairy Sci..

[35] Li P.Y., Sun S., Zhang W.J., Ouyang W., Li X.B., Yang K.L. (2024). The effects of l-citrulline supplementation on the athletic performance, physiological and biochemical parameters, antioxidant capacity, and blood amino acid and polyamine levels in speed-racing yili horses. Animals.

[36] Hristov A.N., Ivan M., Rode L.M., McAllister T.A. (2001). Fermentation characteristics and ruminal ciliate protozoal populations in cattle fed medium-or high-concentrate barley-based diets. J. Anim. Sci..

[37] Wei X.S., Wu H., Wang Z.X., Zhu J.P., Wang W.J., Wang J.H., Wang Y.M., Wang C. (2023). Rumen-protected lysine supplementation improved amino acid balance, nitrogen utilization and altered hindgut microbiota of dairy cows. Anim. Nutr..

[38] Xue B., Zhao X.Q., Zhang Y.S. (2005). Seasonal changes in weight and body composition of yak grazing on alpine-meadow grassland in the Qinghai-Tibetan plateau of China. J. Anim. Sci..

[39] Liu H.S., Ma Z.Y., Pei C.F., Wu D.Z.C.R., Gan S.Y., Zhou J.W. (2024). Comparison of daily gain, rumen fermentation, and blood parameters of fattening yaks under grazing and house feeding patterns. Pratacultural Sci..

[40] Ma W.H., Malik M.I., Iwaasa A.D., Wang H., Wang H.L., Yang J.F., Bai B.Q., Jing J.W., Hu G.W., Hao L.Z. (2025). The Effects of supplemental feeding on methane emissions from yak grazing in the warm season. Animals.

[41] Heiderscheit K.J., Hansen S.L. (2020). Effect of rumen-protected lysine on growth performance, carcass characteristics, and plasma amino acid profile in feedlot steers. Transl. Anim. Sci..

[42] Liu X.J., Yang Z.M., Yang J.F., Wang D.Y., Niu J.Z., Bai B.Q., Sun W., Ma S.K., Cheng Y.F., Hao L.Z. (2023). A comparative study of growth performance, blood biochemistry, rumen fermentation, and ruminal and fecal bacterial structure between yaks and cattle raised under high concentrate feeding conditions. Microorganisms.

[43] Jiang Y.H., Dai P., Dai Q.D., Ma J., Wang Z.S., Hu R., Zou H.W., Peng Q.H., Wang L.Z., Xue B. (2022). Effects of the higher concentrate ratio on the production performance, ruminal fermentation, and morphological structure in male cattle-yaks. Vet. Med. Sci..

[44] Liu J., Tian K., Sun Y., Wu Y., Chen J., Zhang R., He T., Dong G. (2020). Effects of the acid–base treatment of corn on rumen fermentation and microbiota, inflammatory response and growth performance in beef cattle fed high-concentrate diet. Animal.

[45] Wilson P.N., Osbourn D.F. (1960). Compensatory growth after undernutrition in mammals and birds. Biol. Rev..

[46] Yang C., Zhang J.B., Ahmad A.A., Bao P.J., Guo X., Long R.J., Ding X.Z., Yan P. (2019). Dietary energy levels affect growth performance through growth hormone and insulin-like growth factor 1 in yak (*Bos grunniens*). Animals.

[47] Liu Y., Liu J.H., Hao L.Z., Sun P., Degen A. (2023). Effect of substituting steam-flaked corn for course ground corn on in vitro digestibility, average daily gain, serum metabolites and ruminal volatile fatty acids, and bacteria diversity in growing yaks. Anim. Feed Sci. Technol..

[48] Wang H.C., Long R.J., Liang J.B., Guo X.S., Ding L.M., Shang Z.H. (2011). Comparison of nitrogen metabolism in yak (*Bos grunniens*) and indigenous cattle (*Bos taurus*) on the Qinghai-Tibetan Plateau. Asian-Australas. J. Anim. Sci..

[49] Jing X.P., Ding L.M., Zhou J.W., Huang X.D., Degen A.A., Long R.J. (2022). The adaptive strategies of yaks to live in the Asian highlands. Anim. Nutr..

[50] Ji H.Y., Chen L.L., Ma Y., Degen A.A., Yuan Z.R., Chen H.L., Zhou J.W. (2024). A comparison of growth performance, blood parameters, rumen fermentation, and bacterial community of Tibetan sheep when fattened by pasture grazing versus stall feeding. Microorganisms.

[51] Puppel K., Kuczyńska B. (2016). Metabolic profiles of cow’s blood; a review. J. Sci. Food Agric..

[52] Stewart G.S., Smith C.P. (2005). Urea nitrogen salvage mechanisms and their relevance to ruminants, non-ruminants and man. Nutr. Res. Rev..

[53] Zou S.Y., Ji S.K., Xu H.J., Wang M.Y., Li B.B., Shen Y.Z., Li Y., Gao Y.X., Li J.G., Gao Y.F. (2023). Rumen-protected lysine and methionine supplementation reduced protein requirement of Holstein bulls by altering nitrogen metabolism in liver. Animals.

[54] Wang B., Mi M.M., Zhang Q.Y., Bao N., Pan L., Zhao Y., Qin G.X. (2021). Relationship between the amino acid release kinetics of feed proteins and nitrogen balance in finishing pigs. Animal.

[55] Sun Z.H., Tan Z.L., Liu S.M., Tayo G.O., Lin B., Teng B., Tang S.X., Wang W.J., Liao Y.P., Pan Y.F. (2007). Effects of dietary methionine and lysine sources on nutrient digestion, nitrogen utilization, and duodenal amino acid flow in growing goats. J. Anim. Sci..

[56] Tan Z.L., Murphy M.R. (2004). Ammonia production, ammonia absorption, and urea recycling in ruminants. A review. J. Anim. Feed Sci..

[57] Cheng F.X., Jia S.B., Zhang H., Tuo N., Yan X.H., Shao W., Yu X. (2009). Study on the relationship between 15 kinds of serum biochemical and body weight change of warm season grazing lambs. China Anim. Husb. Vet. Med..

[58] Socha M.T., Putnam D.E., Garthwaite B.D., Whitehouse N.L., Kierstead N.A., Schwab C.G., Ducharme G.A., Robert J.C. (2005). Improving intestinal amino acid supply of pre-and postpartum dairy cows with rumen-protected methionine and lysine. J. Dairy Sci..

[59] EClinpath (2020). Glucose.

[60] Ou Y.L., Lai Y.R., Jiang C.N., Zhang J., Ding Z. (2020). Diagnostic performance of individual characteristics and anthropometric measurements in detecting elevated serum alanine aminotransferase among children and adolescents. BMC Pediatr..

[61] Merck R. (1991). The Merck Veterinary Manual.

[62] Shikata N., Maki Y., Noguchi Y., Mori M., Hanai T., Takahashi M., Okamoto M. (2007). Multi-layered network structure of amino acid (AA) metabolism characterized by each essential AA-deficient condition. Amino Acids.

[63] Bach A., Huntington G.B., Calsamiglia S., Stern M.D. (2000). Nitrogen metabolism of early lactation cows fed diets with two different levels of protein and different amino acid profiles. J. Dairy Sci..

[64] Arshad U., Peñagaricano F., White H.M. (2024). Effects of feeding rumen-protected lysine during the postpartum period on performance and amino acid profile in dairy cows: A meta-analysis. J. Dairy Sci..

[65] Lee C., Lobos N.E., Weiss W.P. (2019). Effects of supplementing rumen-protected lysine and methionine during prepartum and postpartum periods on performance of dairy cows. J. Dairy Sci..

[66] Shen Y.Z., Ding L.Y., Chen L.M., Xu J.H., Zhao R., Yang W.Z., Wang H.R., Wang M.Z. (2019). Feeding corn grain steeped in citric acid modulates rumen fermentation and inflammatory responses in dairy goats. Animal.

[67] Van Soest P.J. (1994). Nutritional Ecology of the Ruminant.

[68] Russell J.B., O’Connor J.D., Fox D.G., Van Soest P.J., Sniffen C.J. (1992). A net carbohydrate and protein system for evaluating cattle diets: I. Ruminal fermentation. J. Anim. Sci..

[69] Preston T.R., Leng R.A. (1987). Matching Ruminant Production Systems with Available Resources in the Tropics and Sub-Tropics.

[70] Russell J.B., Sniffen C.J., Van Soest P.J. (1983). Effect of carbohydrate limitation on degradation and utilization of casein by mixed rumen bacteria. J. Dairy Sci..

[71] Tu R. (2024). Effects of Rumen-Protected Lysine in Different Protein Diets on Growth Performance, Nutrient Digestion, Serum Biochemistry, Rumen Fermentation and Microflora Composition of Yaks. Master’s Thesis.

[72] Zhou Z.M., Fang L., Meng Q.X., Li S.L., Chai S.T., Liu S.J., Schonewille J.T. (2017). Assessment of ruminal bacterial and archaeal community structure in yak (*Bos grunniens*). Front. Microbiol..

[73] Kong F.L., Gao Y.X., Tang M.Q., Fu T., Diao Q.Y., Bi Y.L., Tu Y. (2020). Effects of dietary rumen–protected Lys levels on rumen fermentation and bacterial community composition in Holstein heifers. Appl. Microbiol. Biotechnol..

[74] Shannon P., Markiel A., Ozier O., Baliga N.S., Wang J.T., Ramage D., Amin N., Schwikowski B., Ideker T. (2003). Cytoscape: A software environment for integrated models of biomolecular interaction networks. Genome Res..

[75] Loor J.J., Elolimy A.A., McCann J.C. (2016). Dietary impacts on rumen microbiota in beef and dairy production. Anim. Front..

[76] Liu H., Ran T., Zhang C.F., Yang W.Z., Wu X.K., Degen A., Long R.J., Shi Z.J., Zhou J.W. (2022). Comparison of rumen bacterial communities between yaks (*Bos grunniens*) and Qaidam cattle (*Bos taurus*) fed a low protein diet with different energy levels. Front. Microbiol..

[77] de Souza M.G., Reis I.A., de Carvalho I.P.C., de Felicio Porcionato M.A., Prados L.F., Granja-Salcedo Y.T., Siqueira G.R., de Resende F.D. (2022). Effects of post-ruminal urea supplementation during the seasonal period on performance and rumen microbiome of rearing grazing Nellore cattle. Animals.

[78] Zhao F.F., Yang L.L., Zhang T., Zhuang D.H., Wu Q.F., Yu J.K., Tian C., Zhang Z.G. (2023). Gut microbiome signatures of extreme environment adaption in Tibetan pig. NPJ Biofilms Microbiomes.

[79] Méheust R., Castelle C.J., Matheus Carnevali P.B., Farag I.F., He C., Chen L.X., Amano Y.K., Hug L.A., Banfield J.F. (2020). Groundwater Elusimicrobia are metabolically diverse compared to gut microbiome Elusimicrobia and some have a novel nitrogenase paralog. ISME J..

[80] Zheng H., Dietrich C., Radek R., Brune A. (2016). *Endomicrobium proavitum*, the first isolate of *Endomicrobia* class. nov. (phylum *E lusimicrobia*)—An ultramicrobacterium with an unusual cell cycle that fixes nitrogen with a Group IV nitrogenase. Environ. Microbiol..

[81] Alves K.L.G.C., Granja-Salcedo Y.T., Messana J.D., de Souza V.C., Ganga M.J.G., Colovate P.H.D., Kishi L.T., Berchielli T.T. (2021). Rumen bacterial diversity in relation to nitrogen retention in beef cattle. Anaerobe.

[82] Bach A., Calsamiglia S., Stern M.D. (2005). Nitrogen metabolism in the rumen. J. Dairy Sci..

[83] Van Gylswyk N.O., Van der Toorn J.J.T.K. (1985). *Eubacterium uniforme* sp. nov. and *Eubacterium xylanophilum* sp. nov., fiber-digesting bacteria from the rumina of sheep fed corn stover. Int. J. Syst. Evol. Microbiol..

[84] Lang L., Ditton A., Stanescu A., Jainani V., McArthur S., Pourtau L., Gaudout D., Pontifex M.G., Tsigarides J., Steward T. (2025). A standardised saffron extract improves subjective and objective sleep quality in healthy older adults with sleep complaints: Results from the gut-sleep-brain axis randomised, double-blind, placebo-controlled pilot study. medRxiv.

